# Levoglucosan and Its Isomers as Markers and Biomarkers of Exposure to Wood Burning

**DOI:** 10.3390/toxics13090742

**Published:** 2025-08-31

**Authors:** Boglárka S. Balogh, Zsófia Csákó, Zoltán Nyiri, Máté Szabados, Réka Kakucs, Norbert Erdélyi, Tamás Szigeti

**Affiliations:** 1 National Center for Public Health and Pharmacy, Albert Flórián út 2-6, 1097 Budapest, Hungary; baloghboglarkasara@gmail.com (B.S.B.); csako.zsofia@nngyk.gov.hu (Z.C.); szabados.m@met.hu (M.S.); kakucs.reka@nngyk.gov.hu (R.K.); erdelyi.norbert@nngyk.gov.hu (N.E.); 2 Doctoral School of Environmental Sciences, Eötvös Loránd University, Pázmány Péter stny. 1/A, 1117 Budapest, Hungary; 3 HungaroMet—Air Quality Reference Center, Gilice tér 39, 1181 Budapest, Hungary

**Keywords:** human biomonitoring, levoglucosan, wood smoke, exposure biomarkers, urine

## Abstract

Levoglucosan and its isomers, mannosan and galactosan, are widely used atmospheric tracers of biomass combustion, and levoglucosan has been previously proposed as a potential biomarker of wood smoke exposure. This study evaluated their applicability under real-world conditions. During 14-day monitoring campaigns in both heating and non-heating seasons, daily PM_2.5_ and paired urine samples were collected from adults and children in two Hungarian settlements with different heating practices. Monosaccharide anhydrides in PM_2.5_ and urine were quantified by gas chromatography–mass spectrometry, while demographic, dietary, and lifestyle data were obtained via questionnaires. Ambient concentrations were substantially higher during the heating season and at the rural site, confirming the significant contribution of residential wood burning to air pollution. While urinary levoglucosan was quantifiable in >90% of samples, its isomers were often below the limit of quantification. Urinary levoglucosan concentrations did not exhibit consistent seasonal or spatial patterns and were not associated with ambient levels. Instead, an unexplained background more likely influenced by certain demographic, dietary, and behavioral factors than by environmental exposure appeared to drive urinary levels. These findings suggest that urinary levoglucosan is not a suitable biomarker for assessing residential wood smoke exposure, with similar conclusions drawn for mannosan and galactosan.

## 1. Introduction

Air pollution is a leading environmental risk to public health, contributing significantly to respiratory and cardiovascular morbidity and mortality worldwide. In many regions, particularly during winter, residential biomass burning—including wood combustion for heating and cooking—continues to be a major source of particulate matter (PM) and a contributor to elevated PM_2.5_ mass concentrations [1,2,3].

To assess the contribution of biomass combustion to air pollution, several specific chemical tracers have been identified. Among them, levoglucosan (1,6-anhydro-β-D-glucopyranose) and its isomers, mannosan and galactosan, are commonly used markers generated during the thermal degradation of cellulose, and they serve as widely applied chemical tracers for biomass combustion in environmental studies [4,5]. Levoglucosan, in particular, is considered a reliable tracer due to its high abundance and longer stability under diverse environmental conditions [6]. However, beyond their role as tracers of biomass combustion, the emissions and concentration ratios of levoglucosan, mannosan, and galactosan are influenced by several factors. For instance, secondary measures such as oxidation catalysts can substantially reduce their emissions [7], while levoglucosan-to-mannosan ratios vary markedly between different fuels (e.g., hardwoods and pellets), thereby complicating source apportionment [8].

Levoglucosan has also been proposed as an effective biomarker of human exposure to wood smoke [9,10,11]. It is excreted largely unmetabolized in urine, with peak concentrations typically observed within the first few hours (approximately within 3–6 h) after exposure and returning to baseline within 24 h, based on both animal and human studies [9,10,12]. Urinary levoglucosan is therefore of interest in human biomonitoring studies as an indicator of short-term exposure to biomass-derived air pollution.

Human biomonitoring provides valuable information on internal exposure, integrating multiple exposure sources and routes [13,14,15]. Urine is frequently used as a biological matrix in human biomonitoring due to its non-invasive collection, relatively high analyte concentrations, and sensitivity to short-term exposures. Several other compounds, including methoxyphenols [16,17,18] and 1-hydroxypyrene [19], have also been investigated as potential biomarkers of wood smoke exposure, but levoglucosan remains one of the most widely studied [9,10,11,20,21,22]. To the best of our knowledge, mannosan and galactosan have not previously been evaluated as urinary biomarkers.

However, findings from previous studies on the applicability of urinary levoglucosan as a potential biomarker have been inconsistent. While some have reported elevated urinary levoglucosan levels in individuals exposed to biomass smoke [9,11,20], others have found no significant or consistent associations [10,21,23]. Moreover, dietary sources—such as caramelized sugars or smoked foods—have been identified as potential confounders that can influence urinary levoglucosan levels, raising concerns about its specificity as a biomarker of wood smoke exposure [12,24]. Despite its potential, there is currently no standardized framework for interpreting urinary levoglucosan concentrations in real-world exposure contexts, and knowledge remains limited as to its kinetics and the impact of confounding factors.

Motivated by regional concerns and prior ambient monitoring data [25], this study is the first to assess urinary levoglucosan and its isomers as biomarkers of wood smoke exposure in Hungary, where residential biomass combustion contributes substantially to air pollution during the heating season. Our objectives were (i) to evaluate whether urinary levels reflect residential wood smoke exposure in two Hungarian settlements during the heating season and (ii) to examine the influence of certain environmental, demographic, and dietary factors on urinary levoglucosan levels. Importantly, this is also the first study to investigate mannosan and galactosan as potential urinary biomarkers of wood smoke exposure. Through this approach, we sought to contribute to the understanding of levoglucosan and its isomers’ applicability in human biomonitoring and to support evidence-based strategies for reducing population-level exposure to biomass smoke.

## 2. Materials and Methods

### 2.1. Ambient Air Quality Monitoring

Ambient air quality was investigated in two Hungarian settlements with different residential heating practices. Details of the sampling sites have been reported previously [25]. The rural site, Nógrádmegyer (48°04′07″ N, 19°37′26″ E), is a small village of around 1700 residents situated in a valley within the hilly northern region, characterized by widespread wood burning, often combined with illegal waste burning. The urban site, Esztergom (47°47′08″ N, 18°44′25″ E), has approximately 27,000 inhabitants and lies along the Danube River, where most households rely on district heating and natural gas, although residential wood burning remains present to some extent. Sampling was conducted concurrently in both settlements during two periods: the heating season (27 January–9 February 2020) and the non-heating season (26 August–9 September 2020).

Fourteen PM_2.5_ samples were collected at each site during both the heating and non-heating season onto quartz fiber filters (Ø 150 mm, Whatman QM-A, Cytiva, Maidstone, UK) by two Digitel DHA-80 high-volume aerosol samplers (Digitel Elektronik AG, Volketswil, Switzerland) equipped with PM_2.5_ cut-off inlets. Each sampler was loaded with 14 filters, which were automatically changed every 24 h at 6 PM. The average flow rate was maintained at approximately 30 m^3^/h. PM_2.5_ mass concentrations were determined by gravimetric method, weighing each filter before and after sampling on an analytical balance with a readability of 0.1 mg. Before sampling, filters were wrapped in aluminum foil and pre-treated at 550 °C in a laboratory furnace for 8 h in order to eliminate any possible organic contaminants. Before and after sampling, the filters were conditioned for 48 h at 20 ± 1 °C and 50 ± 5% relative humidity. Samples were then stored in glass Petri dishes sealed with parafilm at −20 °C until analysis.

Three stereoisomers of monosaccharide anhydrides, levoglucosan, mannosan, and galactosan, were analyzed using gas chromatography–mass spectrometry (GC-MS) after trimethylsilylation. Briefly, filter punches were extracted twice with acetone, filtered, and evaporated to dryness under nitrogen. Derivatization was performed with N-Methyl-N-(trimethylsilyl)-trifluoroacetamide (MSTFA) containing 1% trimethylchlorosilane (TMCS) in pyridine at 80 °C for 60 min. GC-MS analysis was carried out on an Agilent system equipped with an Rxi-5Sil MS column, using helium as carrier gas and operating in selected ion monitoring mode. The analytical procedure has been described in detail elsewhere [25].

### 2.2. Human Biomonitoring Study

#### 2.2.1. Study Population

The study participants were selected based on the following criteria: (i) the parent, preferably the mother, or legal guardian was over 20 years of age; (ii) the child was between 5 and 14 years old at the start of the survey; (iii) the parent or legal guardian and the child had permanent residence in the selected settlements and had been living together at their current address for at least six months; (iv) selected participants were recruited as adult—child pairs; preferably, neither adults without children nor children without a parent or legal guardian participated; (v) neither the adult nor the child had a diagnosed urinary tract disease, catheter, or any condition preventing urine sample collection; (vi) participants did not intend to relocate in the near future; (vii) preference was given to non-smoking adults.

The characteristics of the study population, including age, gender, smoking habits, and body mass index, are summarized in Table 1. This study included children aged 5 to 13 years and one of their parents or legal guardians, allowing comparison of exposure levels between children and adults. A total of 192 volunteers participated in the study, with 93 residing in the urban site and 99 in the rural settlement. In six cases, only an adult participated in the study without the involvement of a child. Among adults, 15 were male (15.2%) and 84 female (84.8%), while among children, 53 were boys (57.0%), and 40 were girls (43.0%). Additional characteristics of study participants are provided in Table 1. The study protocol was approved by the Medical Research Council of Hungary (registration number: 49631-2/2019/EKU). All volunteers were informed about the study’s purpose, and the parents or legal guardians of each child were required to provide written informed consent for their own and their child’s participation in the study. All data were pseudonymized.

#### 2.2.2. Urine Sample Collection

First-morning urine samples were collected from the volunteers during two sampling campaigns, once in the heating season and once in the non-heating season, each aligned with a 14-day ambient air monitoring period. This design ensured that urine samples were obtained on different days throughout the monitoring period, in parallel with daily PM_2.5_ sampling, to allow for the assessment of temporal variability and potential associations between urinary levoglucosan levels and levoglucosan concentrations measured in PM_2.5_. After signing the informed consent, participants received sample containers and detailed instructions. They were asked to provide a urine sample on the scheduled day of the home visit. Samples were collected in 100 mL polypropylene containers and kept cool during transport in a cool box. Upon arrival, the samples were aliquoted into 10 mL polypropylene tubes and stored at −80 °C until analysis. In total, 380 urine samples were collected across the two monitoring campaigns.

#### 2.2.3. Determination of Levoglucosan, Mannosan, and Galactosan Concentrations in Urine

Standards of levoglucosan, mannosan, and galactosan were purchased from Toronto Research Chemicals (Toronto, ON, Canada). Deuterated levoglucosan (1,6-anhydro-β-D-glucopyranose-d7, 98%), used as an internal standard during extraction, was obtained from Cambridge Isotope Laboratories (Tewksbury, MA, USA). The silylation mixture consisted of 1% (*v*/*v*) trimethylchlorosilane (TMCS) in N-Methyl-N (trimethylsilyl) trifluoroacetamide (MSTFA). Pyridine (silylation solvent), MSTFA, and TMCS were obtained from Sigma-Aldrich (St. Luis, MO, USA). Ethanol and methanol were obtained from Merck KGaA (Darmstadt, Germany), acetonitrile from Avantor/VWR (Leuven, Belgium), and acetone from Thermo Fisher Scientific Inc. (Leicestershire, UK); all solvents were of analytical grade. Ultrapure water was produced in-house using a Millipore RiOs Water Purification System (Merck KGaA, Darmstadt, Germany).

Homogenized urine samples (100 µL) were transferred into Eppendorf tubes, followed by the addition of 50 µL of internal standard solution. After vortexing for 20 s, 900 µL of ethanol was added to precipitate proteins, and the samples were centrifuged at 14,000 rpm for 6 min at 4 °C [5]. The resulting supernatant was transferred into a 4 mL glass vial. Samples were lyophilized in a freeze dryer (Modulyo 4K, Edwards, Crawley, UK) for a minimum of 4 h until completely dry. The remaining contents of the vial were extracted with 1 mL of 98:2 (*v*/*v*) acetonitrile/water mixture in an ultrasonic bath for 15 min. The extraction was repeated twice, and then the extracts were combined. The extract was subjected to solid-phase extraction (SPE) using a 500 mg aminopropyl SPE column (ISOLUTE NH2, 500 mg/6 mL, Biotage, Uppsala, Sweden). The column was preconditioned with 6 mL of methanol, followed by conditioning with 3 mL of a 98:2 (*v*/*v*) acetonitrile/water mixture. After this, the sample extract was loaded onto the column. The column was then washed with 6 mL of acetonitrile and dried under vacuum [21]. Analytes were eluted with 3 mL of methanol and evaporated to dryness under nitrogen in a heated water bath. For silylation, 100 µL of pyridine and 100 µL of MSTFA containing 1% TMCS was added. The mixture was vortexed for 20 s and incubated at 80 °C for 60 min. Derivatized samples were transferred into 1.5 mL vials and stored at −20 °C in a freezer until analysis. Calibration standards were prepared with varying concentrations of monosaccharide anhydrides and a fixed concentration of levoglucosan-d7 in acetone. The standard solutions were evaporated to dryness, derivatized, and subsequently analyzed. Calibration curves consisting of nine points covered the concentration range of 0.4–10 µg/mL for levoglucosan and 0.2–2.5 µg/mL for both galactosan and mannosan.

The samples were analyzed on an Agilent 7890B GC with an Agilent 7000C triple quadrupole mass spectrometer (Agilent Technologies Inc., Santa Clara, CA, USA). Chromatographic separation was performed on a Restek Rxi-17SIL MS column (0.25 mm ID × 60 m length × 0.25 μm film thickness). The GC was operated in split mode with a 10:1 split ratio and helium carrier gas (purity of 6.0) at a flow rate of 1.5 mL/min. A 1 μL aliquot of each sample was injected. The inlet temperature was maintained at 300 °C, and the auxiliary transfer line was set to 310 °C. The following temperature program was used for the GC oven: initial temperature of 60 °C held for 0.1 min, ramped at 40 °C/min to 190 °C, then at 4 °C/min to 215 °C, followed by a final ramp of 45 °C/min to 320 °C, which was held for 2 min. The total run time was 10.5 min.

Quantification was performed using internal standard matrix calibration. Qualitative identification was based on retention times and peak ratios of selected ions. For levoglucosan and mannosan, the following transitions were used: *m/z* 333 to 171 and *m/z* 333 to 103. For galactosan, the transitions were *m*/*z* 333 to 103 and *m*/*z* 317 to 147, as its fragmentation patterns are distinct. For levoglucosan-d7, the transitions were *m*/*z* 339 to 177 and from *m*/*z* 339 to 105. The limit of detection (LOD) and limit of quantification (LOQ) values varied between the three compounds. For the injected solutions, the LOD and LOQ values were 0.025 and 0.4 µg/mL for levoglucosan, 0.025 and 0.2 µg/mL for mannosan, and 0.05 and 0.2 µg/mL for galactosan. For the urine samples, the LOD and LOQ values were 0.05 and 0.8 µg/mL for levoglucosan, 0.05 and 0.4 µg/mL for mannosan, and 0.1 and 0.4 µg/mL for galactosan. Based on an average urinary creatinine concentration of 10.61 mmol/L, the creatinine-adjusted LOD and LOQ values were also calculated. At the beginning of each analytical batch, a blank sample was analyzed. Quality control samples were measured after every tenth sample and at the end of the sequence to verify instrument performance. The validation of the analytical method is provided in the Supplementary Materials (Tables S1–S4). Levoglucosan, mannosan, and galactosan concentrations (µg/mL) in urine were normalized to creatinine levels and expressed as μmol/mol creatinine.

Urinary creatinine concentrations were determined using the Jaffe method [26]. Briefly, a colored complex was formed between creatinine and alkaline picrate and then measured using flow injection analysis with an Agilent 1100 HPLC system equipped with a diode array detector (Agilent Technologies Inc., Santa Clara, CA, USA).

### 2.3. Questionnaire Survey

On the day of urine sample collection, parents completed two paper-based questionnaires with assistance from the study team. The first, titled “Living environment and health,” collected sociodemographic information, details of the living environment (including the use of wood combustion sources such as heating systems, water heaters, and cooking appliances), smoking habits, and health-related data. The second, the “Human biomonitoring” questionnaire, focused on dietary intake within the 24 h prior to urine collection.

### 2.4. Statistical Analyses

Statistical analyses were conducted using IBM SPSS Statistics, version 22.0 (IBM Corporation, Armonk, NY, USA). Concentrations below the LOQ were substituted with half the LOQ value [27]. No missing values were present in the variables used, although not all participants provided samples in both seasons. A *p*-value ≤ 0.05 was considered statistically significant. Descriptive statistics were calculated for all study variables. Urinary levoglucosan levels were summarized using the median, arithmetic and geometric means, standard deviation, 5th and 95th percentiles, and minimum and maximum values. Descriptive comparisons were stratified by key covariates: season (heating vs. non-heating), residential area (rural vs. urban), parental smoking (yes/no), age group (children/adults), gender, domestic wood heating (yes/no), and caramel-flavored food consumption (yes/no). The distribution of creatinine-adjusted urinary levoglucosan concentrations was tested using the Shapiro–Wilk test (*p* < 0.001), indicating a significant deviation from normality. Therefore, non-parametric Mann–Whitney U tests were used to compare group differences across seasons and between population subgroups. False discovery rate correction was applied using the Benjamini–Hochberg procedure. Both uncorrected and adjusted *p*-values are reported. Seasonal differences in urinary levoglucosan concentrations were assessed using the Wilcoxon signed-rank test among participants who provided paired samples during both the heating and non-heating seasons, including stratified analyses by study site, age group, and gender.

## 3. Results and Discussions

### 3.1. Air Quality

#### 3.1.1. PM_2.5_ Mass Concentrations

PM_2.5_ mass concentrations measured in the two settlements have been reported previously [25]. During the heating season, daily mean PM_2.5_ concentrations ranged from 8.9 to 63.4 µg/m^3^ at the rural site (median: 24.5 µg/m^3^) and from 3.1 to 30.9 µg/m^3^ at the urban site (median: 15.3 µg/m^3^). Median PM_2.5_ concentrations during the heating season were approximately twice as high as those measured during the non-heating season at both sites, with ratios of 2.2 in Nógrádmegyer and 1.8 in Esztergom.

The WHO 24 h guideline value for PM_2.5_ (15 µg/m^3^) was exceeded in 79% of the samples in Nógrádmegyer and in 50% of the samples in Esztergom during the heating season. In the non-heating season, exceedances occurred in only 22% of the samples in Nógrádmegyer.

#### 3.1.2. Levoglucosan, Mannosan, and Galactosan Concentrations in PM_2.5_

**Figure 1:**
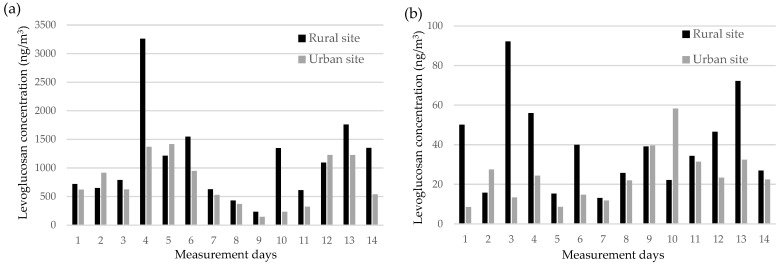
Temporal variation in levoglucosan concentrations in PM_2.5_ at the rural and urban sites during (**a**) the heating and (**b**) the non-heating seasons.

During the heating season, levoglucosan was the most abundant monosaccharide anhydride at both sites, followed by mannosan and galactosan. Median levoglucosan concentrations were substantially higher at the rural site (942 ng/m^3^; range: 235–3262 ng/m^3^) compared to the urban site (624 ng/m^3^; range: 146–1417 ng/m^3^), indicating an approximate 1.5-fold difference. The elevated levels in the rural area are likely attributable to the more frequent use of wood for residential heating. Levoglucosan concentrations were consistently and significantly higher than the combined levels of mannosan and galactosan—typically by an order of magnitude—confirming its dominance as a tracer of biomass burning. In the non-heating season, levoglucosan concentrations were markedly lower at both sites (median: 36.7 and 23.0 ng/m^3^ for the rural and urban sites, respectively), suggesting minimal influence from biomass combustion (Figure 1). Seasonally, levoglucosan levels were approximately 30 times higher during the heating season than in the non-heating season at the rural site, and about 20 times higher at the urban site. Mannosan and galactosan followed a similar seasonal trend, with concentrations remaining far below heating season levels. These trends underscore the strong impact of residential wood burning on monosaccharide anhydride concentrations during the heating season and highlight the importance of seasonal stratification when interpreting spatial differences in exposure [25].

This seasonal pattern is consistent with findings from other studies investigating wood burning (e.g., [28,29]). The mean levoglucosan concentration at the urban site is comparable to values reported in other European cities (e.g., [29,30,31,32,33,34,35,36,37]), while the mean value for the rural site during the heating season exceeds levels observed in comparable rural areas. This is likely due to the continued use of wood for both heating and cooking. Spearman’s rank correlation showed a strong positive association between PM_2.5_ and levoglucosan concentrations (rural site: r_s_ = 0.95, *p* < 0.01; urban site: r_s_ = 0.85, *p* < 0.01), reinforcing the link between biomass combustion and elevated PM_2.5_ levels, particularly in winter. Similarly strong correlations were found for mannosan (r_s_ = 0.92, *p* < 0.01) and galactosan (r_s_ = 0.94, *p* < 0.01), further supporting their role as tracers of biomass burning.

### 3.2. Urinary Concentrations of Monosaccharide Anhydrides

Urine samples were collected from 192 participants across the two campaigns at the study sites. Four participants provided only a single sample during the heating season and did not participate in the sampling during the non-heating season. Levoglucosan was detected in all urine samples and quantified in over 90% (90.5%) of them. Mannosan and galactosan were also measured; however, for mannosan, 58.7% of samples were below the LOQ and 8.7% were below the LOD, respectively, while for galactosan, 41.6% were below the LOQ and 16.8% were below the LOD. Summary statistics for urinary levoglucosan concentrations are shown in Table 2.

The median creatinine-adjusted urinary levoglucosan concentration across all samples was 2.52 µg/mg creatinine. Participants from the rural site had slightly higher median concentrations than those from the urban site, but the difference was not statistically significant (2.77 vs. 2.45 µg/mg creatinine). In contrast, a significant difference was observed between age groups. In both sampling periods, urinary levoglucosan concentrations were significantly higher in children than in adults (median: 3.38 vs. 2.17 µg/mg creatinine).

Paired urinary levoglucosan concentrations from participants who provided samples in both seasons (*n* = 188) were compared using the Wilcoxon signed-rank test. Stratified analyses by age group, location, and sex indicated no significant variation between seasons. Among rural adults, concentrations tended to be higher during the heating season, but the difference was not statistically significant. No seasonal differences were observed in rural or urban children, nor in analyses further stratified by sex within these groups. Detailed subgroup results are provided in Table S5.

**Figure 2:**
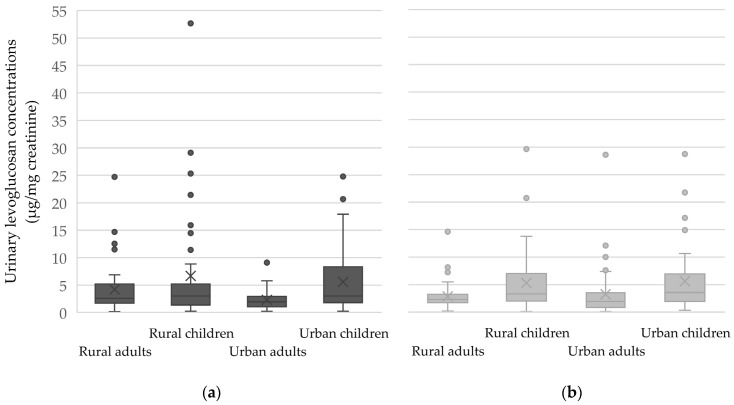
Boxplots of urinary levoglucosan concentrations (μg/mg creatinine) during the heating (**a**) and non-heating seasons (**b**), stratified by residential area and age group. Inner squares in each box correspond to the mean value. The bottom, middle, and top of each box correspond to the 25th, 50th, and 75th percentile, while whiskers indicate 10th and 90th percentiles, and individual outliers are shown as well.

Overall, median creatinine-adjusted concentrations were slightly elevated during the heating season across all subgroups (Figure 2). However, no statistically significant seasonal differences were observed between the heating and non-heating seasons in any subgroup (all *p*-values > 0.05). This may reflect both the substantial variability and the relatively small sample sizes in certain subgroups. Importantly, it may also highlight the limitations of levoglucosan as a robust biomarker of seasonal wood smoke exposure in real-world settings.

Despite the lack of statistical significance, the data suggest a tendency toward higher urinary levoglucosan levels during the heating season among rural adults (Figure 2). In rural children, however, the median concentrations were slightly higher during the non-heating season, suggesting that factors other than residential heating may have influenced their exposure. No clear seasonal differences were observed among urban participants. The boxplots also highlight the wide variability and presence of outliers, particularly among rural children, which may contribute to the lack of statistical significance.

The median creatinine-adjusted urinary concentrations of galactosan and mannosan were approximately an order of magnitude lower than those of levoglucosan (Table 2). Participants from the rural site had slightly higher median concentrations of both compounds than those from the urban site in both seasons, but the differences were not statistically significant. Paired urinary concentrations of galactosan and mannosan between the heating and non-heating seasons were compared using the Wilcoxon signed-rank test. The results are shown in Table S5. For both compounds, concentrations were generally higher during the non-heating season across most subgroups, with statistically significant differences observed in all groups except rural children for mannosan. Urinary galactosan and mannosan levels exhibited an opposite seasonal trend compared to ambient concentrations, suggesting that their presence in urine is influenced by factors other than biomass smoke exposure. These findings indicate that galactosan and mannosan are not suitable biomarkers.

#### 3.2.1. Association Between the Concentrations of Levoglucosan in PM_2.5_ and in Urine

To assess the potential association between levoglucosan concentrations in PM_2.5_ and in urine, Spearman’s rank correlation tests were performed. The analysis was conducted separately by season, location, and age group to evaluate differences within each subgroup. Daily PM_2.5_ levoglucosan concentrations—measured over 24 h periods from 6:00 p.m. on the day prior to urine collection to 6:00 p.m. on the day of collection—were matched to the corresponding urinary levoglucosan concentrations.

No significant associations were observed at either site in either season (all *p* values > 0.3). When stratified by age group, correlations remained weak and non-significant for adults in both seasons. A moderate negative association was observed among rural children during the non-heating season (r_s_ = −0.57, *p* = 0.051), suggesting a possible but not statistically confirmed inverse relationship.

These findings indicate that daily variations in levoglucosan concentrations in PM_2.5_ were not reflected in urinary levoglucosan concentrations. This weak trend in children could be influenced by other factors. The detailed results for all subgroups are provided in Table S6.

#### 3.2.2. Impact of Different Factors on Urinary Levoglucosan Concentration

Several factors may influence urinary levoglucosan concentrations. We analyzed the impact of selected variables, including age group, gender, residential area, smoking habits, heating type, green waste burning, and caramel-flavor food consumption. The detailed results are summarized in Table 3. In addition to comparing the overall study population, we also separately evaluated whether urinary levoglucosan concentrations differed between the heating and non-heating seasons in children and in adults, in order to account for their distinct exposure patterns and physiological characteristics.

Age group. Urinary levoglucosan concentrations were significantly higher in children compared to adults. The median creatinine-adjusted urinary levoglucosan concentration was 3.38 µg/mg creatinine in children and 2.13 µg/mg creatinine in adults. A similar pattern was observed by Wallner et al. [11]. This difference may reflect physiological characteristics such as children’s higher breathing rate per body weight, smaller body size, higher metabolic rate, and immature organ systems [38,39,40], as well as behavioral and dietary factors that may also contribute. This pattern is also common for other biomarkers (e.g., pesticides, bisphenols, and phthalates) and may reflect differences in physiology or diet [41]. Therefore, the consistently higher urinary levoglucosan in children—even during non-heating seasons—does not necessarily indicate higher exposure to wood smoke.

Gender. A significant difference was also observed between male and female participants, with a median creatinine-adjusted urinary levoglucosan concentration of 3.54 µg/mg creatinine in males and 2.30 µg/mg creatinine in females. This difference may reflect metabolic differences, behavioral factors, or dietary habits, although the underlying mechanisms remain unclear and warrant further investigation. When stratified by age, gender-related differences were consistently significant among adults during both the heating and non-heating seasons. In contrast, among children, a significant difference was observed only during the heating season, while no difference was found during the non-heating season. These findings indicate that gender-related variability in urinary levoglucosan is more pronounced in adults than in children. Similar observations were reported by [23], who found that male participants tended to have higher urinary levoglucosan levels even prior to exposure, suggesting that factors unrelated to biomass smoke (such as diet or higher breathing rates) may also contribute to the observed differences. Pairwise comparisons further showed that boys had higher urinary levoglucosan concentrations than girls in both seasons, although this difference cannot be conclusively attributed to wood smoke exposure.

Residential area. Participants living in rural areas had slightly higher median creatinine-adjusted urinary levoglucosan concentrations (2.77 µg/mg creatinine) compared to urban residents (2.43 µg/mg creatinine), although this difference was not statistically significant. Wallner et al. [11] reported similar trends in Austria, where the pooled urine samples of rural regions showed higher levoglucosan concentrations. Although not significant in our study, these results suggest that local heating practices and ambient air quality may influence exposure levels. When stratified by age, a significant rural–urban difference was observed among adults during the heating season (*p* = 0.005), with rural adults showing higher concentrations. In contrast, no significant differences were detected among children in either season. These findings suggest that the impact of residential area on urinary levoglucosan concentrations is limited to adults during the heating season, while children’s exposure appears unaffected by whether they live in rural or urban settings.

Smoking habits. Tobacco smoke is a potential source of levoglucosan [42,43,44], and evidence suggests that levoglucosan is primarily excreted within a few hours, whereas cotinine—the main biomarker of tobacco exposure—has a half-life of 16–19 h [45]. During the heating season, 15 of the 99 adult samples came from active smokers. Median urinary levoglucosan concentrations were identical among smokers and non-smokers (2.17 µg/mg creatinine), suggesting no clear difference in exposure. This lack of association may be due to the relatively small number of smokers and the fact that all reported smokers were women, who generally had lower levoglucosan levels than men. Moreover, smoking status was based solely on self-report, which may have led to exposure misclassification, as under-reporting of smoking is plausible. Similar issues were reported by Migliaccio et al. [9], where parent-reported smoking in the home was associated with urinary levoglucosan, but biomarker-based assessment was not, likely reflecting differences in pharmacokinetics between levoglucosan and cotinine as well as potential misreporting of tobacco exposure. Among children, median concentrations were slightly higher in those living in non-smoking households (3.35 µg/mg creatinine) than in those exposed to household smoking (2.65 µg/mg creatinine), but the difference was not statistically significant. These findings suggest that residential biomass burning has a more pronounced impact on urinary levoglucosan levels than tobacco smoke, although potential under-reporting of smoking could have obscured the true differences.

Heating with wood. Wood stoves were the primary heating source in 42.7% of the investigated households. In adults, median urinary levoglucosan concentrations were slightly higher among participants living in homes with wood stoves compared to those without, although the difference was not statistically significant. In children, the median concentration was slightly lower in households with wood stoves, and this difference was also not significant. While previous studies, such as Migliaccio et al. [9], reported higher (non-significant) levels in children living in homes with wood stoves, our findings did not confirm this trend.

Green waste burning. Based on questionnaire responses, 111 of 188 participants, including both adults and children, were living in households burning green waste (e.g., leaves, branches) during the non-heating season. This information reflects habitual behavior rather than actual burning during the sampling days. Interestingly, participants who reported this habit had slightly lower median urinary levoglucosan concentrations (2.53 µg/mg creatinine) compared to those who did not report burning green waste (3.88 µg/mg creatinine), though the difference was not statistically significant. Since green waste burning contributes to higher PM_2.5_ mass concentrations, its potential impact on both children and adults during the non-heating season cannot be ruled out, despite the small and non-significant differences observed.

Caramel-flavor food consumption. Dietary intake was another variable with a measurable impact. Caramel-flavored food or drink consumption within 24 h prior to sampling was reported by 35 samples, originating from 22 children and 13 adults, each of whom provided two urine samples—once during each sampling period. The median creatinine-adjusted urinary levoglucosan concentration among caramel consumers was 4.79 µg/mg creatinine, compared to 2.50 µg/mg creatinine in non-consumers, representing a statistically significant difference. Although previous studies reported a rapid and transient increase in urinary levoglucosan—peaking within 2 h after caramel ingestion [10]—it is uncertain whether our morning urine samples captured this short-lived effect, especially when consumption may have occurred earlier than the evening prior to sampling. Nevertheless, when stratified by age, the effect of caramel consumption remained significant among adults in both the heating and non-heating seasons, with caramel consumers showing consistently higher urinary levoglucosan concentrations. Among children, although the median concentrations were also higher in caramel consumers, the differences were not statistically significant. These results suggest that the observed association with caramel-flavored food consumption is more pronounced in adults than in children, but the timing of ingestion relative to urine collection may have influenced the findings. This is consistent with findings showing that orally administered levoglucosan causes a rapid but short-lived increase in urinary levels, returning to baseline within hours [12]. This supports the assumption that the timing of caramel intake strongly affects its detectability in urine samples.

### 3.3. Comparison with Other Studies

Urinary levoglucosan has been proposed as a biomarker for wood smoke exposure, as it is a specific and stable tracer of biomass combustion in ambient PM_2.5_ [9,11]. However, human studies have produced conflicting results due to rapid excretion, dietary confounding, and differences in study design, leaving its reliability uncertain.

Migliaccio et al. [9] examined both an animal model (mice) and a small group of human participants consisting of 14 children. In mice, wood smoke exposure significantly increased urinary levoglucosan, supporting its potential in controlled settings. In humans, levoglucosan was detectable in all urine samples, but the children’s exposure to wood smoke could not be clearly confirmed. Mean concentrations were slightly higher in children living in homes with wood stoves, but the difference was not significant. The study reported indoor and outdoor PM_2.5_ and particle-phase levoglucosan levels but did not collect dietary or smoking data.

Wallner et al. [11] investigated mothers and their children in rural and urban Austria. Concentrations were consistently higher in rural participants, likely reflecting more frequent biomass use. In all communities, pooled urine samples from children contained higher levels than those from their mothers. However, the use of pooled samples is a major limitation, as it masks individual variability. No environmental PM or particle-phase levoglucosan measurements were reported.

Hinwood et al. [23] monitored 12 wildland firefighters (mean age: 40 years; 9 males, 3 females) during controlled land fire operations in Australia. Urinary levoglucosan was measured in individual pre- and post-shift samples, but no significant differences were observed despite documented smoke exposure. The study did not include dietary or smoking information, which could have influenced the results.

Similarly, Bergauff et al. [10] conducted two controlled wood smoke exposure trials using smoke generated from an older-model wood stove. Four non-smoking men (18–65 years) participated in the first trial, with spot urine collected before and at four time points after exposure. In the second trial, the same four men plus one woman were included, with an additional sample collected the next morning. Despite elevated PM_2.5_ and particle-phase levoglucosan, urinary concentrations did not consistently increase. In a separate dietary study, they showed that caramel ingestion caused a rapid but short-lived urinary peak at 2 h, decreasing by 12 h, and returning to baseline within 24 h in most participants.

Naeher et al. [20] collected 97 pairs of pre- and post-shift urine samples from 19 firefighters over 10 shifts. Although mean levels increased significantly after shifts, individual responses varied, with some showing no change or decreases, suggesting additional influencing factors despite high PM_2.5_ exposure.

A more recent study by Navarro et al. [22] confirmed the link between high-intensity exposure and urinary levoglucosan levels in wildland firefighters. While they observed an increase in post-shift concentrations (for instance, pre-shift median 9.7 µg/mg creatinine vs. post-shift 47 µg/mg creatinine), the results also highlighted the biomarker’s rapid clearance and temporal variability. This further underscored the challenges of using urinary levoglucosan to assess more diffuse, community-level exposure.

Sankaranarayanan et al. [21] assessed urinary levoglucosan in children before and after indoor air quality interventions. Despite substantial PM_2.5_ reductions, urinary levels did not decrease, and no significant association with indoor PM_2.5_ was observed.

Two studies also highlighted dietary impacts. Hinwood et al. [23] found small but measurable increases in four out of twelve participants who consumed vanilla or clove-flavored foods during sampling, while Bergauff et al. confirmed short-term peaks after caramel intake [10]. Tobacco smoke, another potential source, showed no clear association with urinary levels in previous studies or in our own findings, possibly due to rapid excretion or under-reporting of smoking. These findings are further supported by an oral administration study, where 15 mg of levoglucosan resulted in urinary concentrations peaking within 3 h, with approximately 70% of the dose excreted within 7 h [12].

Taken together, these studies show that differences in population characteristics, pooled versus individual sampling, control of diet and smoking, and the rapid elimination of levoglucosan substantially limit its specificity as a urinary biomarker for wood smoke exposure. Key features of these studies are summarized in Table S7.

### 3.4. Limitations of Using Levoglucosan as a Biomarker

While this study investigated the potential of urinary levoglucosan as a biomarker of wood smoke exposure, several limitations reduced its applicability.

First, the most important limitation is the presence of a background level in nearly all urine samples, even during the non-heating season. This was also reported by other studies [12,21,23] and strongly supports the existence of a continuous, unidentified background source. Controlled animal studies have shown that background-free urine can be achieved under strictly managed conditions [9,12], suggesting that in humans the background likely arises from dietary or metabolic sources not investigated in our study. While certain factors have been identified as likely contributors, they do not fully explain the observed levels. The inability to quantify or exclude this background fundamentally limits the specificity of urinary levoglucosan. Further studies should focus on identifying the sources of the background level and on combining levoglucosan with other indicators in well-controlled exposure settings.

Second, levoglucosan has a short biological half-life, but the reported elimination times vary between studies. Controlled oral dosing studies demonstrated that the compound peaks in urine within 3–6 h and is largely excreted within 7–24 h [9,10,12]. Consequently, measurements reflect only very recent exposure, and without precise timing of exposure and sampling, interpretation is uncertain.

Furthermore, while ambient air data provide a useful proxy for community-level exposure, the lack of indoor air quality measurements represents a limitation of our study.

Finally, individual factors such as breathing rate, metabolism, and activity patterns may influence biomarker levels in urine, including levoglucosan, but these effects are inherently difficult to quantify. They may differ between children and adults or between urban and rural residents, yet they appear secondary to the overarching background problem. Without detailed personal exposure and behavior data (e.g., time spent indoors, proximity to burning sources), it remains impossible to disentangle wood smoke exposure from other contributions. Therefore, the main challenge lies not in physiological variability itself but in the inability to clearly separate the influence of residential wood smoke from other sources. While other biomarkers also face specificity challenges, their sources and metabolism are better characterized.

## 4. Conclusions

To the best of our knowledge, this is the first study to comprehensively examine the influence of multiple factors on urinary levoglucosan levels in a large population to evaluate its suitability as a biomarker for residential wood smoke exposure. Based on our findings, urinary levoglucosan is not a reliable source-specific biomarker for estimating individual exposure to emissions from residential solid fuel combustion.

Our findings demonstrate that although levoglucosan was quantifiable in nearly all urine samples (>90%), urinary concentrations did not consistently reflect seasonal and spatial differences in ambient levoglucosan levels. Instead, an unexplained background level shaped by demographic, dietary, and behavioral factors appeared to influence urinary levels more than the measured environmental exposures. The presence of this background—even in samples collected during the non-heating season and in areas with minimal biomass burning—suggest the existence of additional, unidentified sources that cannot be excluded based on current knowledge. Regarding its isomers, mannosan and galactosan, the observed seasonal trends in their urinary concentrations were opposite to those of ambient levels, indicating that they are also unsuitable as biomarkers.

Further studies should focus on clarifying the origin of the background levels, better characterizing the kinetics of levoglucosan excretion in humans, and exploring a combined approach using other candidate biomarkers (e.g., methoxyphenols, 1-hydroxypyrene) that better capture exposure to solid fuel combustion.

## Figures and Tables

**Table 1:** Characteristics of the studied population.

Variables	All Study Participants (*n* = 192)	Participants at the Rural Site (*n* = 99)	Participants at the Urban Site (*n* = 93)
Age group:			
Adults, *n* (%)	99 (51.6%)	52 (52.5%)	47 (50.5%)
Children, *n* (%)	93 (48.4%)	47 (47.5%)	46 (49.5%)
Age (years):			
Adults, mean (min, max)	40 (21–68)	37 (21–68)	42 (31–59)
Children, mean (min, max)	10 (5–13)	10 (5–13)	9 (6–13)
Gender:			
Male, *n* (%)	63 (32.8%)	33 (33.3%)	30 (32.3%)
Female, *n* (%)	129 (67.2%)	66 (66.7%)	63 (67.7%)
Smoker (adults), *n* (%)	15 (15.2%)	13 (25.0%)	2 (4.3%)
Body mass index (kg/m^2^):			
Adults, mean (SD)	25.3 (±5.5)	27.3 (±4.6)	23.3 (±5.7)
Children, mean (SD)	18.2 (±4.1)	18.9 (±4.5)	17.5 (±3.6)

Abbreviations: *n*: number of cases, SD: standard deviation.

**Table 2:** Summary statistics for urinary levoglucosan, mannosan, and galactosan concentrations measured across both heating and non-heating seasons among the 192 participants.

	Levoglucosan	Galactosan	Mannosan
	Creatinine-Adjusted Concentrations (µg/mg Creatinine)		
Number of samples	*n* = 380		
Minimum	0.19	0.02	0.02
5th percentile	0.59	0.04	0.03
Median	2.52	0.35	0.25
Arithmetic mean (SD)	4.48 (±0.35)	0.52 (±1.40)	0.40 (±0.70)
Geometric mean (95% CI)	2.77 (2.51–3.05)	0.18 (0.16–0.21)	0.20 (0.18–0.23)
95th percentile	15.40	1.25	1.17
Maximum	52.67	26.08	11.81

Abbreviations: SD: standard deviation, CI: confidence interval.

**Table 3:** Comparison of urinary levoglucosan concentrations (µg/mg creatinine) between groups defined by age group, gender, residential area, smoking habits, heating type, green waste burning, and caramel-flavored food consumption, based on median values and Mann–Whitney U test (uncorrected and adjusted *p*-values).

Factor	Number of Samples (*n*)	Median Urinary Levoglucosan Concentration (µg/mg Creatinine)	Mann–Whitney Test Heating Season Uncorrected *p*-Value (Adjusted *p*-Value)	Mann–Whitney Test Non-Heating Season Uncorrected *p*-Value (Adjusted *p*-Value)
Age group				
- Adult	196	2.13	*p* < 0.05	*p* < 0.001
- Children	184	3.38	(0.044)	(<0.001)
Gender				
- Male	125	3.54	0.311	*p* < 0.001
- Female	255	2.30	(0.533)	(<0.001)
Residential area				
- Rural site	198	2.77	0.089	0.814
- Urban site	182	2.43	(0.214)	(0.888)
Smoking (only adults)				
- No	166	2.17	0.768	0.924
- Yes	30	2.17	(0.922)	(0.924)
Wood stove in home (only heating season)				
- No	82	2.57	0.733	-
- Yes	110	2.46	(0.977)	
Green waste burning (only non-heating season)				
- No	77	3.88	-	0.410
- Yes	111	2.53		(0.615)
Consumption of caramel-containing food				
- No	245	2.50	*p* < 0.05	0.190
- Yes	35	4.79	(0.063)	(0.380)

## Data Availability

Data will be made available on request.

## Supplementary Materials

The following supporting information can be downloaded at: https://www.mdpi.com/article/10.3390/toxics13090742/s1, Table S1: Selectivity/specificity results obtained for galactosan, mannosan, and levoglucosan; Table S2: Accuracy and precision obtained for galactosan, mannosan, and levoglucosan using urine samples and standard solutions at three concentration levels (*n* = 7 for each); Table S3: Limit of detection and limit of quantification values obtained for galactosan, mannosan, and levoglucosan; Table S4: Sample stability; Table S5. Comparison of paired urinary monosaccharide anhydride concentrations between heating and non-heating periods using the Wilcoxon signed-rank test; Table S6. Spearman’s rank correlation between daily PM_2.5_ levoglucosan concentrations and urinary levoglucosan concentrations, stratified by season, location, and age group; Table S7. Comparison of findings from previous studies.
