# Supplementary material for: Levoglucosan and Its Isomers as Markers and Biomarkers of Exposure to Wood Burning
Source: Toxics. 2025 Aug 31;13(9):742. doi: 10.3390/toxics13090742 (PMC12473716; doi:10.3390/toxics13090742)
Supplement: Supplementary file 1 [file toxics-13-00742-s001.zip › toxics-3832600-supplementary.pdf]

Supplementary materials

I. Method validation

Selectivity / specificity

To evaluate the specificity of the method, a method blank (prepared without urine), a calibration standard, unspiked urine sample, and urine sample spiked with the analytes were analyzed for levoglucosan, mannosan, and galactosan, using levoglucosan-d7 as the internal standard. The absence of interfering peaks in the blanks and the agreement between measured and expected concentrations in spiked samples confirmed that the method is specific for the target analytes. Chromatographic evaluation confirmed that no interfering peaks were observed at the retention times of the analytes in the method blanks.

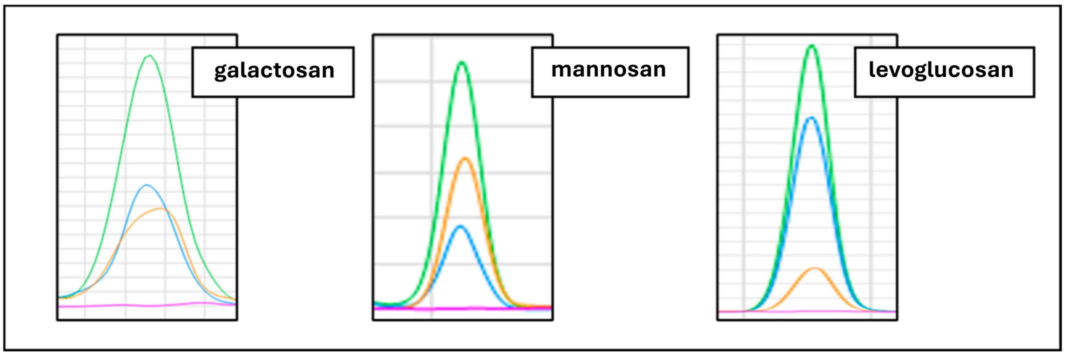

The concentrations measured in the spiked samples were consistent with expected values, while the small amounts naturally present in unspiked urine did not cause any quantitative error. As the observed differences were consistent with the validation criteria, the method was confirmed to be specific for levoglucosan, mannosan, and galactosan. Table S1 shows the measured concentrations of the analytes in method blanks, unspiked urine, and spiked samples, confirming that only the expected analytes were detected under the tested conditions.

Table S1. Selectivity/specificity results obtained for galactosan, mannosan, and levoglucosan

Compound	Method blank (ng/mL)	Unspiked urine (ng/mL)	Spiked urine (200 ng/mL)	Calibration point (200 ng/mL)
Galactosan	-	203	464	200
Mannosan	-	109[1]	312	200
Levoglucosan	-	696	932	198

[1] Extrapolated estimate, value below the limit of quantification.

Accuracy and precision

Accuracy and precision were evaluated using spiked human urine samples at low, medium, and high concentrations of the analytes, as summarized in Table S2.

Table S2. Accuracy and precision obtained for galactosan, mannosan, and levoglucosan using urine samples and standard solutions at three concentration levels (n=7 for each)

		Galactosan	Mannosan	Levoglucosan
200 / 400 ng/mL	Mean (ng/mL)	198.3	185.6	416.4
	Recovery (%)	*99.2*	*92.8*	*104.1*
	Precision (%RSD)	*18.8*	*11.7*	*11.9*
2500 ng/mL	Mean (ng/mL)	2336	2529	2984
	Recovery (%)	*93.4*	*101.2*	*119.4*
	Precision (%RSD)	*4.9*	*1.8*	*4.0*
10000 ng/mL	Mean (ng/mL)	-	-	9737
	Recovery (%)	-	-	*97.4*
	Precision (%RSD)	-	-	*3.2*

Abbreviation: RSD: relative standard deviation.

Linearity

For galactosan and mannosan, the working range was 200–2500 ng/mL; for levoglucosan, it was 400–10000 ng/mL. The relative errors (%RE) of the back-calculated concentrations for all calibration points were below 10%, and all values remained under the 20% acceptance limit. These results confirm that the method is linear across the tested concentration ranges.

Limit of detection (LOD) and limit of quantification (LOQ)

The LOD is the lowest concentration where the signal-to-noise ratio is at least 3:1. LOD values were determined by measuring standard solutions at low concentrations. As no blank urine sample completely free of the target analytes was available, the evaluation of LOD was based solely on the instrumental signal-to-noise criterion. The determined LOD values are presented in Table S3.

The LOQ is the lowest concentration of analyte that can be quantitatively determined with acceptable precision (RSD < 25%) and accuracy (80–120%). In addition, under normal conditions, the blank concentration plus six times the standard deviation of the blank should not exceed this concentration. However, since no urine matrix completely free of the analytes was available, this criterion could not be applied. The lowest tested concentration that fulfilled the above requirements was 400 ng/mL. The LOQ values determined for the analytes are summarized in Table S3.

Table S3. Limit of detection and limit of quantification values obtained for galactosan, mannosan, and levoglucosan

Compound	Limit of detection (ng/mL)	Limit of quantification (ng/mL)
Galactosan	50	200
Mannosan	25	200
Levoglucosan	25	400

Sample stability

Analytes may degrade during preparation, extraction, and storage; therefore, post-preparative stability was investigated. The short-term stability of levoglucosan, mannosan, and galactosan in human urine was examined at −20°C. Samples spiked at 2500 ng/mL were stored and remeasured after 5 and 10 days using calibration performed on the measurement day. Samples were considered stable if concentrations remained within 80–120% and %RSD < 25% (Table S4). All analytes met these criteria, confirming stability under the tested conditions.

Table S4. Sample stability.

**Compound**	**Day**	**$C_{measured}$ (mean) (ng/mL)** 2500 ng addition (n=7)	**Recovery (%)**	**Precision (%RSD)**
Levoglucosan	0	2336	93.4	4.9
	5	2459	98.4	8.5
	10	2474	98.9	4.9
Galactosan	0	2336	93.4	4.9
	5	2459	98.4	8.5
	10	2474	98.9	4.9
Mannosan	0	2529	101.2	1.8
	5	2555	102.2	5.5
	10	2530	101.2	5.8

II. Results of the statistical analysis

Table S5. Comparison of paired urinary monosaccharide anhydride concentrations between heating and non-heating periods using the Wilcoxon signed-rank test

	Group	Participants (n)	Median urinary concentration (µg/mg creatinine) (heating period vs. non-heating period)		p-value
Levoglucosan	Rural adults	52	2.56	2.29	0.088
	Rural children	47	3.00	3.33	0.615
	Urban adults	44	1.96	1.95	0.401
	Urban children	45	3.23	3.57	0.737
	Children - female	38	2.48	2.40	0.803
	Children - male	53	3.89	4.63	0.562
	Rural children - female	17	2.46	2.35	0.795
	Urban children - female	21	2.51	2.50	0.903
	Rural children - male	30	3.98	4.57	0.478
	Urban children - male	23	3.82	4.91	0.927
Galactosan	Rural adults	52	0.37	0.61	<0.001
	Rural children	47	0.19	0.39	<0.001
	Urban adults	44	0.22	0.45	0.006
	Urban children	45	0.22	0.39	0.002
Mannosan	Rural adults	52	0.25	0.35	<0.001
	Rural children	47	0.19	0.26	0.111
	Urban adults	44	0.21	0.35	<0.001
	Urban children	45	0.15	0.30	0.003

Table S6. Spearman's rank correlation between daily $PM_{2.5}$ levoglucosan concentrations and urinary levoglucosan concentrations, stratified by season, location, and age group

Subgroup	r_s	p-value
Heating period - rural - adults	–0.18	0.572
Heating period - rural - children	–0.10	0.762
Non-heating period - rural - adults	–0.29	0.366
Non-heating period - rural - children	–0.57	0.051
Heating period - urban - adults	–0.03	0.929
Heating period - urban - children	–0.13	0.697
Non-heating period - urban - adults	–0.22	0.471
Non-heating period - urban - children	–0.22	0.471

Table S7. Comparison of findings from previous studies

Study	Population (sex/age)	Sample size (n)	Urine samples	Exposure route and PM	Caramel tracked	Smoking	Association of LEV with wood smoke/PM exposure	Excretion time	Notes
**Hinwood et al., 2008**	Firefighter trainees (mixed adults)	12	Individual	Inhalation (training); PM not measured	No	Non-smoking subjects	No significant increase in LEV in 2 h post-exposure	Only single spot data within 2 h of fighting the fire	Single spot sampling limits interpretation; low sensitivity
**Migliaccio et al., 2009**	Mice (Balb/c), children (pilot)	~11 mice; small child cohort	Individual	Intranasal instillation and inhalation of lab PM (mice) and wood stove in homes (children)	No	Not relevant for mice; association with reported parent smoking, but not urinary cotinine	Significant for mice Not significant for children	Mice: ~4 h peak, ~70% excretion; children: ~24 h detectable	Exposure misclassification likely due to under-reporting of smoking; rural poverty context important
**Bergauff et al., 2010**	Adult volunteers (mixed)	9 + 4	Individual	Campfire and wood stove smoke exposure in controlled settings; personnel $PM_{2.5}$ monitors	Experimental caramel consuming	Non-smoking subjects	No consistent response to wood smoke exposure	~12 h,	Experimental study; LEV did not consistently increase despite increasing $PM_{2.5}$
**Moshammer et al., 2012**	Self-experiment	2	Individual	Oral dosing (5 mg LEV); no PM data	Not relevant	Not relevant	-	Peak ~3 h; ~70% dose excreted by 7 h; half-life ~4–5 h	Provides clear pharmacokinetics; not representative for inhalation exposure
**Naeher et al., 2013**	Wildland firefighters (adults, mixed)	19 wildland firefighters over 10 prescribed burns	Individual	Real-world inhalation; personal $PM_{2.5}$, CO measured	No	18 non-smoker and one smoker subject	No consistent response to wood smoke exposure	Post-exposure increase in 63% of paired samples; kinetics not specified	High variability, suggesting other sources
**Wallner et al., 2013**	Austrian communities — mothers and children	5 different type of communities, 10 families in each	Pooled	Ambient residential exposure; PM not directly measured	No	Cotinine in pooled samples; passive exposure noted.	LEV consistently higher in rural participants (not significant)	Not reported	Community-level design; exposure differences assessed via agrarian quota.

**Sankaranarayanan et al., 2016**	Children (boys/girls, residential)	33 children before and after the installation of an air filtration unit in homes	Individual	Inhalation (wood stoves in homes); indoor $PM_{2.5}$ monitored	No	Home smoking noted, not fully excluded	No significant correlation between changes in indoor $PM_{2.5}$ concentrations and changes in urinary LEV concentrations	Kinetics not analyzed	High inter- and intra-individual variability of LEV
**Navarro et al., 2023**	Wildland firefighters (mixed adults)	19	Individual	Inhalation of wildfire smoke and chainsaw exhaust; VOCs, aldehydes, BTEX, and naphthalene measured; PM not directly reported	No	Not specified	65% of paired samples showed post-shift increase in urinary levoglucosan; largest increase on day 3 during mop-up	LEV half-life ~4.5 h; increases observed within single shift	Paired pre- and post-shift urine samples (55 pairs over 3 days); only 3 days on one fire.

Abbreviation: LEV: levoglucosan
